# Update of liver fibrosis and steatosis with transient elastography (Fibroscan)

**DOI:** 10.1093/gastro/got007

**Published:** 2013-03-08

**Authors:** Grace Lai-Hung Wong

**Affiliations:** ^1^Institute of Digestive Disease, The Chinese University of Hong Kong, Hong Kong SAR, China ^2^Department of Medicine and Therapeutics, The Chinese University of Hong Kong, Hong Kong SAR, China

**Keywords:** cirrhosis, hepatitis, fatty liver, histology, liver biopsy, liver stiffness measurement

## Abstract

**Background:** Assessment of liver fibrosis and steatosis is now almost indispensable in most of the chronic liver diseases in order to determine prognosis and need for treatment, and to monitor disease progression and response to treatment. Liver biopsy is limited by its invasiveness and patient acceptability. Transient elastography (TE; Fibroscan) is a non-invasive tool with satisfactory accuracy and reproducibility to estimate liver fibrosis.

**Aims & Methods:** To review the existing evidence concerning the clinical applications of TE in major liver diseases, including chronic hepatitis B and -C, non-alcoholic fatty liver disease (NAFLD), alcoholic liver disease, primary biliary cirrhosis and primary sclerosing cholangitis.

**Results:** As alanine aminotransferase (ALT) is one of the major confounding factors of liver stiffness in chronic hepatitis B, an ALT-based algorithm has been developed and higher liver stiffness measurements (LSM) cut-off values for different stages of liver fibrosis should be used in patients with elevated ALT levels up to five times the upper limit of normal. Furthermore, falsely-high LSM results up to the cirrhotic range may occur during ALT flare. TE is also useful predicting patient prognosis in the development of hepatocellular carcinoma (HCC), portal hypertension, postoperative complications in HCC patients and survival. Unfortunately, failed acquisition of TE is common in obese patients. Furthermore, obese patients may have higher LSM results, even in the same stage of liver fibrosis. To better evaluate NAFLD a new XL probe, with a larger probe with lower ultrasound frequency and deeper penetration, increases the success rate of TE in obese patients. The median LSM value with the XL probe was found to be lower than that by the conventional M probe, hence cut-off values were approximately 1.2 to 1.3 kilopascals lower than those of the M probe, suggesting its adoption. Studies reveal that a novel ultrasonic controlled attenuation parameter is potentially useful to detect and quantify hepatic steatosis non-invasively.

**Conclusion:** TE is a non-invasive, accurate and reproducible test of liver fibrosis and possibly hepatic steatosis and has been validated in a wide spectrum of liver diseases. TE is also useful to predict patient outcomes.

## INTRODUCTION

Liver fibrosis is the natural wound-healing response to parenchymal injury in chronic liver diseases. It may eventually result in liver cirrhosis and its various complications. Accurate staging of liver fibrosis is now essentially indispensable in the decision process for treatment in chronic viral hepatitis, as well as disease prognosis [1, 2]. It is also vital to monitor disease progression and response to treatment.

## LIVER BIOPSY: IS IT STILL A ‘GOLD STANDARD’ ASSESSMENT OF LIVER FIBROSIS?

Liver biopsy has been the ‘gold standard’ for assessing liver fibrosis in the last few decades [3]. However it has numerous limitations, namely its invasive nature, risk of complications, patient discomfort and sampling errors [4]. Complications associated with liver biopsy are rare but can be severe and even life-threatening. Pain and hypotension are the predominant complications for which patients are hospitalized [5]. Clinically significant intraperitoneal hemorrhage is the rarest but most serious bleeding complication of percutaneous liver biopsy, which may happen more often in older-aged patients with cirrhosis or liver cancer [6]. The mortality rate among patients after percutaneous liver biopsy is approximately 1 in 10 000 to 1 in 12 000 [7]. All these problems make it impractical to perform serial biopsies to assess disease progression in routine clinical practice [2].

The diagnostic accuracy of liver biopsy is limited by sampling variability. The average size of biopsy is 15 mm in length, which represents 1/50 000 the size of the entire liver. There is significant variability in the histological assessment of two readings of the same biopsy by the same pathologist and between two pathologists, even among those who are highly specialized [4]. This variability is low for the diagnosis of cirrhosis (kappa coefficient of concordance ≥0.80), moderate for earlier fibrosis stages (kappa 0.70–0.80) but high for the activity grades (kappa 0.40–0.50) [4].

## THE WORKING PRINCIPLES OF TRANSIENT ELASTOGRAPHY

**Figure 1:**
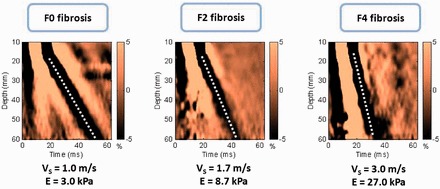
Shear wave propagation velocity according to the severity of hepatic fibrosis (Metavir score). The elastic modulus *E* expressed as *E* = 3*ρV*^2^, where *V* is the shear velocity and *ρ* is the mass density (constant for tissues): the stiffer the tissue, the faster the shear wave propagates. Hence, for absent fibrosis (*F*0), velocity is 1.0 m/s and elasticity is 3.0 kPa, whereas for cirrhosis (*F*4) velocity is 3.0 m/s and elasticity is 27.0 kPa. Modified from Sandrin *et al.* [10].

Transient elastography (TE; Fibroscan®, Echosens, Paris, France) measures liver stiffness in patients suffering from different chronic liver diseases [8, 9]. An ultrasound transducer probe is mounted on the axis of a vibrator. Vibrations of mild amplitude and low frequency (50 Hz) are transmitted by the transducer, inducing a plastic shear wave that propagates through the underlying tissues. Pulse-echo ultrasound acquisition is used to follow the propagation of the shear wave and to measure its velocity, which is directly related to tissue stiffness (the elastic modulus E expressed as E = 3ρV^2^, where V is the shear velocity and ρ is the mass density, which is constant for tissues). The stiffer the tissue, the faster the shear wave propagates (Fig. 1). TE measures liver stiffness in a volume that approximates a cylinder 1 cm in diameter and 4 cm in length, between 25 and 65 mm underneath the skin surface. This volume is at least 100 times bigger than a biopsy sample and therefore should be more representative of the liver parenchyma [8].

TE has the advantages of being painless, rapid (usually less than 5 minutes) and easy to perform at the bedside or in the outpatient clinic. The examination is performed on a non-fasting patient lying supine with the right arm placed behind the head to facilitate access to the right upper quadrant of the abdomen. The tip of the probe transducer is placed on the skin between the rib bones at the level of the right lobe of the liver where liver biopsy would be performed. Once the measurement area has been located, the operator presses the button on the probe to start an acquisition. The software determines whether each measurement is successful or not. Results are expressed in kilopascals (kPa) and correspond to the median of 10 validated measurements according to Sandrin *et al.* [8]. According to the manufacturer, the examination is considered reliable if ≥10 valid measurements are acquired, the success rate (number of valid acquisitions divided by the number of attempts) is over 60%, and the ratio of the interquartile range to the median of 10 measurements (IQR/M) is ≤0.3 [8].

## ACCURACY OF TRANSIENT ELASTOGRAPHY

Reproducibility of TE is an important feature for its widespread clinical application. The reproducibility of liver stiffness measurement (LSM) was excellent for both inter-observer and intra-observer agreement, with intra-class correlation coefficients (ICC) of 0.98 [10]. However, inter-observer agreement was significantly reduced in patients with lower degrees of liver fibrosis (ICC for F0–1 and F2 were 0.60 and 0.99, respectively), with hepatic steatosis (ICC for steatosis <25% and 25% of hepatocytes 0.98 and 0.90, respectively) and with increased body mass index (BMI; ICC for BMI ≥25 kg/m^2^ and <25 kg/m^2^ were 0.98 and 0.94, respectively).

Using TE to assess liver fibrosis has been widely validated in different liver diseases, including chronic hepatitis C (CHC) [1, 11–12], chronic hepatitis B (CHB) [13–15], co-infection with HIV [16], non-alcoholic fatty liver disease (NAFLD) [17–18], alcoholic liver disease [19], primary biliary cirrhosis, primary sclerosing cholangitis [20] and in the post-liver transplantation setting [21]. In these studies, TE was valid with liver histology being the gold standard. In general, all these studies confirm that TE has good overall accuracy to diagnose advanced fibrosis and cirrhosis, independent of the underlying etiology [22–23]. The remaining controversy is the optimal cut-off values to diagnose advanced fibrosis and cirrhosis, which differ according to particular etiologies. This has significant implications when a clinician interprets TE results. The suggested diagnostic performance and cut-off values for histological cirrhosis (F4) based on published studies are summarized in Table 1.

**Table 1.** Diagnostic performance and suggested cut-off values of transient elastography for the diagnosis of histological cirrhosis (F4)

Reference number	[10]	[65]	[66]	[67]	[13]	[15]*	[14]	[40]	[12]	[1]	[11]	[16]	[68]	[69]	[21]	[70]	[17]*	[18]	[19]	[20]
**No. of biopsies**	200	775	354	94	161	238	173	228	251	183	150	72	169	95	124	67	52	246	174	95
**Prevalence of cirrhosis (*F*4; %)**	12.0	15.5	13.3	17.0	25.0	23.5	8.0	20.2	19.0	25.0	19.3	23.6	38.5	17.0	11.0	7.5	5.8	10.1	53.7	16.0
**Etiologies**	All	All	All	All	HBV	HBV	HBV	HCV & HBV	HCV	HCV	HCV	HCV-HIV	HCV-HIV	HCV-LT	HCV-LT	NAFLD	NAFLD	NAFLD	ALD	PBC & PSC
**Proposed cut-off values (kPa)**	11.9	14.6	17.6	16.0	13.4	9.0 (normal ALT)	11.0	14.0	14.6	12.5	14.8	11.8	14.6	12.0	12.5	17.0	10.2	10.3	22.7	17.3
																										12.0 (elevated ALT)														
**Sensitivity (%)**	91	79	77	89	60	54	93	78	86	87	94	100	93	93	100	100	100	92	84	93
**Specificity (%)**	89	95	97	96	93	99	87	98	96	91	92	92.7	88	93	87	98	100	88	83	95
**Negative predictive value (%)**	98	96	92	98	88	67	99	82	97	95	98	82	94	99	100	95	100	99	82	99
**Positive predictive value (%)**	53	74	91	80	75	98	38	98	78	77	73	100	86	74	50	64	100	46	85	78
**Positive LR**	8.3	15.8	25.7	22.3	85	3.3	7.0	39.0	23.1	9.7	11.3	13.7	7.8	14.0	7.7	50.0	∞	7.5	5.24	18.6
**Negative LR**	0.1	0.1	0.2	0.1	0.43	0.7	0.08	0.2	0.1	0.1	0.07	0	0.1	0.1	0	0	0	0.09	0.19	0.1
**AUROC**	0.90	0.95	0.96	0.94	0.93	0.88	0.93	0.96	0.97	0.95	0.99	0.97	0.95	0.90	0.98	0.99	1.00	0.95	0.87	0.96

ALD = alcoholic liver disease, ALT = alanine aminotransferase, AUROC = area under receiver operating characteristics curves, HBV = hepatitis B virus infection,

HCV = hepatitis C virus infection, HCV-HIV = hepatitis B virus and human immunodeficiency virus co-infection, HCV-LT = hepatitis C virus infection recurrence after liver transplantation, LR = likelihood ratio, NAFLD = non-alcoholic fatty liver disease, PBC = primary biliary cirrhosis, PSC = primary sclerosing cholangitis. *Cut-off values proposed for advanced fibrosis (F3 or above).

## CLINICAL APPLICATIONS OF TRANSIENT ELASTOGRAPHY

### Pre-treatment assessment of liver fibrosis

The severity of liver fibrosis is the key factor of timing and choice of therapy. This is particularly relevant in chronic viral hepatitis. Current international guidelines recommend antiviral therapy for CHB patients with significant liver fibrosis [24–26]. As TE has been repeatedly shown to have satisfactory accuracy to exclude and diagnose advanced fibrosis and cirrhosis, as mentioned above, more than half of the patients might reach a treatment decision without the need for confirmatory liver biopsies [13]. TE is also found to be more cost-effective than liver biopsy [27]. TE has been incorporated in the international guidelines for CHB and CHC [24–25].

### Follow-up assessment of liver fibrosis

A few longitudinal studies have reported that patients responding to treatment had low or decreased liver stiffness [28]. In fact, both reduction in fibrosis and necroinflammation might contribute to the decrease in liver stiffness [29]. In a prospective study of 71 CHB patients on antiviral therapy, paired liver biopsy and TE were both performed at baseline and at 1 year following treatment [30]. Although TE remained accurate in distinguishing patients with insignificant disease from those with advanced fibrosis or cirrhosis at both time points, the absolute change in liver stiffness correlated poorly with the change in histological fibrosis stage and resolution of advanced fibrosis could only be assumed with significantly decreased liver stiffness to 5.0 kPa or less after antiviral treatment [30].

### Prediction of portal hypertension and variceal bleeding

TE is found useful to identify cirrhotic patients with higher risk of portal hypertension and cut-off values of 17.6 kPa and 21.0 kPa having sensitivity ≥90%, in order to detect patients with hepatic venous pressure gradient (HVPG) above 10–12 mmHg [31–32]. The presence of varices could be excluded with a liver stiffness below 12.5–19.8 kPa [33–34]. Unfortunately, these suggested cut-off values overlap with those for detecting histological cirrhosis in most chronic liver diseases. Hence there seems to be no significant new information provided by TE regarding screening endoscopy for varices among cirrhotic patients.

### Prediction of hepatocellular carcinoma

TE is also useful in predicting the risk of other liver-related complications and death. A dose–response relationship between LSM and risk of hepatocellular carcinoma (HCC) was found in both CHB and CHC patients (Table 2). Taking patients with LSM ≤10.0 kPa as reference, the hazard ratios of developing HCC were 17, 21, 26 and 46 in patients with LSM at 10.1–15.0 kPa, 15.1–20.0 kPa, 20.1–25.0 kPa and above 25.0 kPa, respectively, in a prospective cohort of 866 CHC patients [35]. Patients with LSM ≤8.0 kPa acted as the control group; the hazard ratios of developing HCC were 3.1, 4.7, 5.6 and 6.6 in patients with LSM at 8.1–13.0 kPa, 13.1–18.0 kPa, 18.1–23.0 kPa, and above 23.0 kPa, respectively, in another cohort of 1,130 CHB patients [36]. LSM, as well as FibroTest, can also predict 5-year survival of patients with CHC; the prognostic values of LSM remained even after adjustments for treatment response, patient age and degree of necroinflammation [37].

**Table 2.** Liver stiffness measurement (LSM) and the risk of hepatocellular carcinoma (HCC) in chronic hepatitis B or C patients [41, 42]

Chronic hepatitis B patients		Chronic hepatitis C patients	
LSM	Hazard ratios of HCC	LSM	Hazard ratios of HCC
≤10.0 kPa	Referent	≤8.0 kPa	Referent
10.1–15.0 kPa	17	8.1–13.0 kPa	3.1
15.1–20.0 kPa	21	13.1–18.0 kPa	4.7
20.1–25.0 kPa	26	18.1–23.0 kPa	5.6
>25.0 kPa	46	>23.0 kPa	6.6

### Prediction of postoperative outcomes

LSM is also an important prognostic tool in patients confirmed to have HCC. A prospective study of 105 HCC patients demonstrated that an LSM cut-off of 12.0 kPa had a sensitivity of 86% and specificity of 72% in prediction of major post-operative complications [38]. This cut-off might also identify patients with more severe operative blood loss and higher transfusion rates [38]. Another study of 133 HCC patients revealed that patients with LSM ≥13.4 kPa had a nearly twofold increase in the risk of HCC recurrence, compared to those with LSM <13.4 kPa [39].

## LIMITATIONS OF TRANSIENT ELASTOGRAPHY

### Factors affecting accuracy of measurements

**Figure 2:**
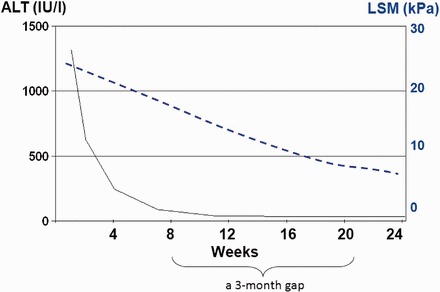
Falsely elevated liver stiffness measurement (LSM) results in a patient with grossly elevated alanine aminotransferase levels. LSM values decreased considerably after the resolution of acute hepatitis. Modified from Wong *et al.* [35].

**Figure 3:**
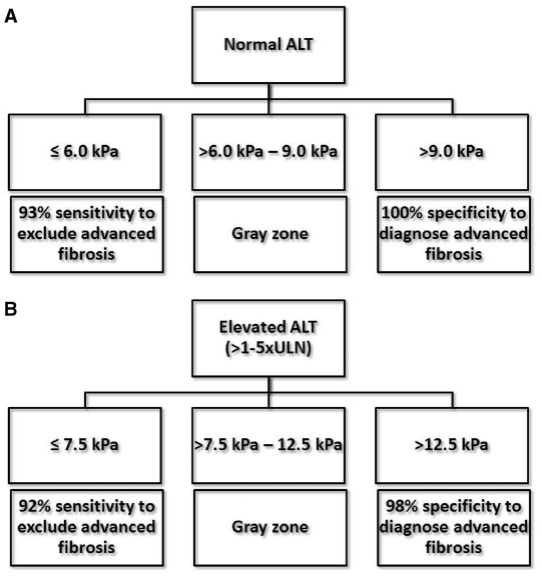
An alanine aminotransferase (ALT)-based algorithm for (A) normal ALT and (B) elevated ALT levels up to five times upper limit of normal to exclude or establish advanced liver fibrosis for chronic hepatitis B patients. Modified from Chan *et al.* [16].

Not only liver fibrosis but also other factors contribute to liver stiffness. LSM has been consistently found to be falsely elevated in acute hepatitis, manifested as alanine aminotransferase (ALT) flares [40–41]. Severe hepatic necroinflammation may lead to LSM values well within the cirrhotic range, even in the absence of fibrosis on histology [29, 42–43]. In this setting, LSM tends to decrease considerably after the resolution of acute hepatitis. Therefore, applying TE in this scenario can be misleading and is not recommended until at least 3 months after normalization, or at least until stabilization of ALT levels below five times the upper limit of normal [13, 41] (Fig. 2). An ALT-based algorithm has been developed and higher LSM cut-off values for different stages of liver fibrosis should be used in patients with elevated ALT levels (Fig. 3).

Extrahepatic cholestasis [44], hepatic congestion [45], hepatic amyloidosis [46] and recent food intake (within 60 minutes) [47] were also found to be associated with a falsely high LSM values. Fortunately, the degree of hepatic steatosis does not appear to affect LSM results: therefore TE remains an accurate tool for fibrosis assessment in CHC and NAFLD [11, 18]. Our recent study showed that NAFLD patients with BMI 30 kg/m^2^, the lowest limit of an abnormal BMI in NAFLD, would have higher LSM values by M probe even in the same fibrosis stage [48]. This provocative finding may lead to concern about the M probe’s accuracy in obese patients. The emergence of the XL probe is a possible solution to this issue.

### Factors affecting success rate of measurements

It has been noted that unreliable and failed LSMs occur, respectively, at about 3% and 11.6–18.4% in all TE examinations and they are independently associated with BMI >30 kg/m^2^ in both Caucasians and Chinese [49–50]. The success rate of LSMs with the M probe would be as low as 75% in NAFLD patients with BMI >30 kg/m^2^ [18]. The low LSM success rate among obese patients is likely related to the thick subcutaneous fat, which hinders the transmission of shear waves and ultrasound waves through the liver parenchyma [50]. Patients with extreme—very high and very low—BMI were recently found to have higher LSM values in an Indian population [51]. Subjects with narrow intercostal space, high-riding liver, hyperinflated lungs, ascites or free peritoneal fluid may also have lower success rate or failed acquisition of LSM [8].

A recent study challenged the validity of the reliability criteria, suggested by the manufacturer, of 1165 patients with chronic liver diseases, who underwent LSM within 3 months of liver biopsy. The investigators found that the number of successful acquisitions, and their success rate, had no influence on the diagnostic accuracy [52]. Furthermore, LSM remained reliable even if the ratio of the interquartile range to the median of 10 measurements (IQR/M) >0.30, provided that the median LSM was below 7.1 kPa. These new findings implied that LSM results were more reliable than what had been previously described.

## COMBINING TRANSIENT ELASTOGRAPHY WITH SERUM MARKERS

In general, serum markers have modest accuracy for diagnosing advanced liver fibrosis [53–54]. TE has certain advantages over serum markers as it provides a more direct measurement of fibrosis, is less affected by intercurrent health disorders and is theoretically applicable to all chronic liver diseases. On the other hand, its diagnostic performance was particularly affected in patients with elevated serum ALT levels [29]; hence a second non-invasive test, independent of the serum ALT or AST levels, may be a good supplementary test for LSM. Among various serum test formulae, the Forns index [55] and Hui index [53] are composed of clinical parameters other than ALT or AST levels. We demonstrated that a combined LSM-Forns algorithm improved the accuracy to predict advanced liver fibrosis in 238 CHB patients [15]. In this combined algorithm, low LSM or low Forns index could be used to exclude advanced fibrosis with a high sensitivity of 95%. To confirm advanced fibrosis, agreement between high LSM and high Forns index could improve the specificity up to 99–100% [15].

The combination of TE and FibroTest was found to have the best diagnostic performance, compared to either test alone, in patients with CHC [1]. When TE and FibroTest matched (present in 70–80% of cases), results were also concordant, respectively, in 84%, 95% and 94% of patients with liver fibrosis ≥F2, ≥F3 and F = 4 [1]. The combination of LSM and FibroTest allowed exclusion of significant fibrosis (≥F2) in nearly 80% of 100 CHB patients in the inactive carrier stage.

## NEW FEATURES OF TRANSIENT ELASTOGRAPHY

### S and XL probes

**Figure 4:**
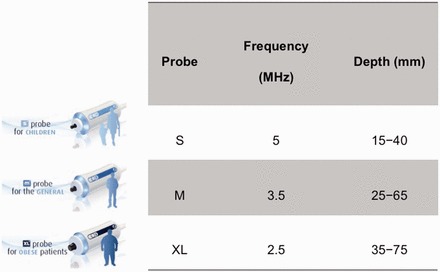
The characteristic of the new S and XL probes comparing to M probe.

The development of S and XL probes aim to cater for different population groups of various body build types (Fig. 4). The S probe contains a higher frequency ultrasonic transducer and shallower measurements below the skin surface, which suit pediatric subjects and those with small body build [56]. The XL probe contains a lower frequency and a more sensitive transducer, a deeper focal length, larger vibration amplitude and a greater depth of measurements below the skin surface [57]. This probe serves obese subjects with ‘XL’ body builds. Data concerning the validations of these new probes are emerging.

With the XL probe, LSM could be successfully performed in more obese patients compared to the M probe [58]. In our validation study involving 286 patients, LSM using the XL probe documented reliable results in 92% of patients, compared to 80% using the M probe [64]. In another study of 193 NAFLD patients, a cut-off value had reasonable sensitivity (78%), specificity (78%), positive predictive value (60%) and good negative predictive value (89%) for F3 or greater disease [59]. However, the median LSM by the XL probe was consistently found to be approximately 1.0 kPa to 1.2 kPa lower than that of the M probe at the same stage of liver fibrosis in all of the histological reports [58–59]. A recent exploratory study of 517 overweight patients having different etiologies, XL cut-off values of 4.8 kPa and 10.7 kPa were the best estimates of 6.0 kPa and 12.0 kPa with the M probe, for patients with BMI > 25-30 kg/m^2^ [48]. Patients with BMI > 30 kg/m^2^ might use M probe cut-offs for the XL probe. More studies are warranted to delineate the proper cut-off values of LSM using the XL probe in various etiologies.

### Controlled attenuation parameter

As obesity is becoming a pandemic and is increasingly encountered worldwide in the last few decades [60], the prevalence of NAFLD has been substantially increased [61]. This makes the estimation of the degree of hepatic steatosis essential. Recently, a novel physical parameter, based on the properties of ultrasonic signals acquired by the Fibroscan machine, has been developed, applying the property that hepatic steatosis affects ultrasound propagation [62]. This novel parameter, ‘controlled attenuation parameter’ (CAP), is measuring ultrasound attenuation at the center frequency (expressed as dB/m) of the M probe. In a recent study of 112 patients with liver biopsy, CAP was found efficient in detecting low grade steatosis [58]. A cut-off value of 215 dB/m has a sensitivity of 90% to detect S1 steatosis [58]. In order to evaluate hepatic steatosis, the data supports the use of CAP simultaneously with LSM. This would be a promising new tool to monitor the development of NAFLD not only in patients with high BMI, but in ‘metabolically obese’ patients, as recent evidence demonstrated that distribution of fat (not total fat) was associated with NAFLD [63–64].

## CONCLUSIONS

TE is a non-invasive, accurate and reproducible test of liver fibrosis—and possibly hepatic steatosis—and has been validated in a wide spectrum of liver diseases. TE is also useful in predicting patient outcomes. Further studies should explore the appropriate cut-off values of newer XL and S probes, as well as those of the novel controlled attenuation parameter (CAP).

**Conflict of interest:** G.W. has served as a speaker for Echosens and an advisory committee member for Otsuka.
